# Consensus criteria for the diagnosis of scabies: A Delphi study of international experts

**DOI:** 10.1371/journal.pntd.0006549

**Published:** 2018-05-24

**Authors:** Daniel Engelman, L. Claire Fuller, Andrew C. Steer, for the International Alliance for the Control of Scabies Delphi panel

**Affiliations:** 1 Group A Streptococcal Research Group, Murdoch Children’s Research Institute, Melbourne, Victoria, Australia; 2 Centre for International Child Health, Department of Paediatrics, University of Melbourne, Melbourne, Victoria, Australia; 3 Department of General Medicine, Royal Children’s Hospital, Melbourne, Victoria, Australia; 4 Chelsea and Westminster Hospital, London, United Kingdom; 5 International Foundation for Dermatology, London, United Kingdom; University of California San Diego School of Medicine, UNITED STATES

## Abstract

**Background:**

Scabies was added to the WHO Neglected Tropical Diseases portfolio in 2017, and further understanding of the disease burden is now required. There are no uniformly accepted test methods or examination procedures for diagnosis, which limits the interpretation of research and epidemiological findings. The International Alliance for the Control of Scabies (IACS) designated harmonization of diagnostic procedures as a priority for the development of a global control strategy. Therefore, we aimed to develop consensus criteria for the diagnosis of scabies.

**Methodology / Principal findings:**

We conducted an iterative, consensus (Delphi) study involving international experts in the diagnosis of scabies. Panel members were recruited through expression of interest and targeted invitation of experts. The Delphi study consisted of four rounds of anonymous surveys. Rounds 1 and 2 involved generation and ranking an extensive list of possible features. In Rounds 3 and 4, participants were presented results from previous rounds and indicated agreement with a series of draft criteria. Panel participants (n = 34, range per Round 28–30) were predominantly highly experienced clinicians, representing a range of clinical expertise and all inhabited continents. Based on initial rounds, a draft set of criteria were developed, incorporating three levels of diagnostic certainty–Confirmed Scabies, Clinical Scabies and Suspected Scabies. Consensus was reached in Round 4, with a very high level of agreement (> 89%) for all levels of criteria and subcategories. Adoption of the criteria was supported by 96% of panel members.

**Conclusions / Significance:**

Consensus criteria for scabies diagnosis were established with very high agreement. The 2018 IACS Criteria for the Diagnosis of Scabies can be implemented for scabies research and mapping projects, and for surveillance after control interventions. Validation of the criteria is required.

## Author summary

Scabies causes rash and severe itch and predisposes to serious infection and chronic diseases of the heart and kidneys. Despite scabies being an ancient disease found in all parts of the world, we currently lack reliable laboratory tests. Clinicians generally make an assessment based on history and skin examination. Variation in diagnosis causes problems when trying to determine the prevalence of scabies in a region, or when trying to investigate the effectiveness of a treatment or control strategy. We aimed to establish consensus among experts on how to diagnose scabies. Thirty-four international experts responded to anonymous questionnaires on how tests and clinical features of scabies should be used to form a diagnosis. Draft criteria were developed and refined, incorporating three levels of diagnostic certainty–Confirmed Scabies, Clinical Scabies and Suspected Scabies. After three rounds of surveys, 82% of participants supported the criteria. After four rounds, consensus was reached with very high agreement for all criteria (Confirmed Scabies, 96%; Clinical Scabies, 93%; Suspected Scabies, 100%) and 96% supported adoption of the criteria. It is hoped the 2018 IACS Criteria for the Diagnosis of Scabies will allow harmonization of diagnosis and reporting, and comparison between studies of scabies burden and treatments.

## Introduction

Scabies is a common skin condition, caused by the ectoparasite *Sarcoptes scabiei* var. *hominis*. The Global Burden of Disease study estimates more than 200 million people are affected at any one time [1]. The direct effects of scabies are estimated to account for 0.21% of DALYs caused by all conditions [2], but the broader impact, incorporating complications of bacterial skin infection, invasive bacterial disease and auto-immune kidney and heart disease is likely to be substantially greater [3].

The World Health Organization has recently designated scabies as a neglected tropical disease (NTD) for large-scale disease control action [4]. Whilst there has been a very high prevalence reported in some population-based studies, there is a paucity of disease burden data from most regions [5]. There are numerous opportunities to integrate population-based surveys for scabies with mapping and surveillance for other NTDs and health programs [6, 7], but few successful examples thus far [8]. Therefore, standardized methods are needed for further epidemiological mapping, and to monitor the effectiveness of control interventions.

The need to improve and standardize the diagnosis of scabies was identified as a priority at the inaugural meeting of the International Alliance for the Control of Scabies (IACS) in Atlanta, 2012 [3]. Microscopy of skin samples has limited sensitivity and utility in field settings, and there are no laboratory tests available. A systematic review of diagnostic methods used in therapeutic trials for scabies found wide variation with no uniformity [9], similar to results of a previous systematic review [10]. Given the limitations in available evidence for diagnostic methods, we aimed to develop consensus criteria for the diagnosis of human scabies in a range of epidemiological and research environments, using a formal consensus process.

## Methods

The Delphi method is an established, iterative, multi-stage process for developing consensus using at least two rounds of anonymous surveys [11]. The Delphi method has been used for establishment of diagnostic and/or treatment frameworks where there is a lack of scientific evidence to make recommendations, including for several skin conditions [12–14]. After identification of the research issue and formation of the panel of participants, initial survey rounds generate a range of ideas and opinions through open-ended questions. In subsequent rounds, participants are provided a summary of the responses of the whole panel, and then given the opportunity to revise their own response. The advantages of the method include the ability to involve a large group of international participants, relatively low cost, anonymity, and reducing the likelihood of dominance by certain participants. Disadvantages include arbitrary cut-offs for consensus, potential for influence of moderators, and length of time required [11, 15].

### Moderators and panel members

The moderator group was comprised of a pediatrician, pediatric infectious disease physician and dermatologist, all experienced in the diagnosis and management of scabies and other tropical skin diseases, and all members of the IACS Steering Committee.

Recruitment to the panel was via an expression of interest circulated to the IACS membership, as well as targeted invitations to experts in scabies and tropical dermatology known to the working group or panel members. Moderators did not participate in the survey rounds. A panel of 10–15 members is usually recommended [15]. We aimed to have at least 20 respondents for each round.

### Agreement

Agreement was defined as the percentage of positive responses (agree or strongly agree) divided by the total completed responses (excluding responses outside areas of expertise). Consensus was defined *a priori* as 65–79% agreement indicating moderate consensus and ≥ 80% agreement indicating strong consensus.

### Delphi survey rounds

The Delphi process consisted of four rounds. In Round 1, participants were asked to contribute all possible features relating to the diagnosis of scabies that could be considered for inclusion in the diagnostic criteria, including features on history, physical examination and investigation methods. Open-ended questions were used. Participants were asked to consider diagnosis in both resource-limited / field settings and high income / office-based settings. This generated a comprehensive list of possible features.

In Round 2, participants were asked to rank the importance of this long list of features for diagnosis using a visual analogue scale (from ‘not at all’ to ‘very important’). Further questions regarding clinical examination, investigations and dermatoscopy were assessed using categorical responses with four possible options (essential, important but not essential, not important, not required). Participants were also encouraged to provide further detail about the proposed list of features, possible combinations of features and the structure of the diagnostic criteria using free text responses. Visual analogue scales were converted to numerical values from 0 to 100. Features with a median value < 60 were omitted, those with value > 80 were retained and those between 60–79 were discussed by the moderator group, alongside all comments, taking into account the known evidence base. The narrowed list of features was developed into a draft set of criteria.

In Round 3, the draft set of criteria was presented, along with the analysis from Round 2. Participants indicated agreement to each aspect of the criteria, as well as the overall structure, using four-point Likert-type questions (strongly disagree, disagree, agree, strongly agree) or had the option of declining to respond if the item was outside their specific area of expertise (for example, questions related to mite visualization techniques). Participants provided additional free-text comments wherever there was disagreement to a criterion, or to provide suggestions about structure, syntax, clarity of phrasing and terminology. The working group further revised the draft criteria based on the analysis of the Round 3 agreement and free text commentary.

In Round 4, the revised criteria were presented, along with the agreement results of Round 3 and explanations for any revisions. Participants were again asked to state their agreement with each aspect of the criteria and the criteria structure, using the same Likert-type questions. Demographic information of participants was also collected in Round 4.

Study data were collected and managed using REDCap electronic data capture tools hosted at Murdoch Children’s Research Institute [16]. Up to three reminders were sent to participants who had not responded. All responses and comments were anonymous. Comments were compiled, grouped by theme and discussed by the moderator group after each round.

## Results

Thirty-four panel participants completed at least one survey. Participants were predominantly highly experienced dermatologists with practice across a range of settings and representing all major geographic regions. The characteristics of the Round 4 participants are shown in Table 1.

**Table 1:** Characteristics of Delphi panel members responding to Round 4 survey.

Characteristic	N	%
Sex		
Female	13	46.4
Male	15	53.6
Occupation		
Dermatologist	25	89.3
Researcher or Academic	2	7.1
Infectious Disease Physician	1	3.6
Experience with diagnosis of scabies, settings*		
Developed settings	14	50.0
Low / middle income settings	22	78.6
Hospitals	22	78.6
Clinics	18	64.3
Field settings	14	50.0
Experience with diagnosis of scabies, years		
7–9	3	10.7
≥ 10	25	89.3
Principal region of current practice		
Africa	4	14.3
Asia	5	17.9
Oceania	3	10.7
Europe	6	21.4
Northern America	5	17.9
Central and South America	5	17.9

*Not mutually exclusive

Responses were received from 30 participants for both Round 1 and Round 2. Participants expressed that criteria should only be used for diagnosis of common scabies, and not crusted or other atypical clinical variants. Based on responses, a set of proposed criteria were developed that could be used in a variety of settings, by incorporating three levels of diagnostic certainty–Confirmed Scabies, Clinical Scabies and Suspected Scabies.

Twenty-eight responses were received for Round 3. Agreement on the structure and contents of the draft criteria was high, with 23 respondents (82%) supporting the adoption of the draft criteria (Table 2). Extensive commentary was also received about content and terminology, leading to further revision of the draft criteria.

**Table 2:** Round 3 survey responses.

	Strongly Disagree	Disagree	Agree	Strongly Agree	Agreement
	n				%
Level A: Confirmed scabies	0	5	10	13	82.1
Level B: Clinical scabies	0	6	11	10	77.8
Level C: Suspected scabies	0	4	14	9	85.2
Support criteria structure	0	1	9	18	96.4
Support criteria adoption	0	5	12	11	82.1

Twenty-eight responses were received for Round 4. There was a high level of agreement on all levels of criteria and individual subcategories (Table 3). Twenty-seven participants (96.4%) supported the adoption of the proposed criteria. Further commentary from panelists resulted in minor editing of terminology to improve clarity.

**Table 3:** Round 4 survey responses.

	Strongly Disagree	Disagree	Agree	Strongly Agree	Agreement
	n				%
Level A: Confirmed scabies	0	1	9	18	96.4
Microscopy	0	1	7	20	96.3
High-powered visualization	0	1	9	17	96.3
Dermoscopy	0	0	9	19	100.0
Level B: Clinical scabies	0	2	11	15	92.9
Burrows	0	0	12	16	100.0
Male genital lesions	0	1	9	17	96.3
Typical lesions and distribution and two history features	0	1	10	17	96.4
Level C: Suspected scabies	0	0	11	17	100.0
Typical lesions and distribution and one history feature	0	0	9	19	100.0
Atypical lesions or distribution and two history features	0	3	8	17	89.3
Support criteria adoption	0	1	9	18	96.4

## Discussion

We established consensus on criteria for the diagnosis of scabies through a four-round Delphi process (Box 1). The final product is a single set of criteria with three levels and eight subcategories, representing a spectrum of diagnostic certainty. The agreement for each level and subcategory was very high, with at least 89% agreement for all subcategories, and 96% support for the adoption of the criteria.

**Box 1. Summary of 2018 IACS criteria for the diagnosis of scabies**

**A: Confirmed scabies**

**At least one of**:
A1: Mites, eggs or feces on light microscopy of skin samples
A2: Mites, eggs or feces visualized on individual using high-powered imaging device
A3: Mite visualized on individual using dermoscopy

**B**: **Clinical scabies**

**At least one of**:
B1: Scabies burrows
B2: Typical lesions affecting male genitalia
B3: Typical lesions in a typical distribution and two history features

**C**: **Suspected scabies**

**One of**:
C1: Typical lesions in a typical distribution and one history feature
C2: Atypical lesions or atypical distribution and two history features

**History** **features**

H1: Itch
H2: Close contact with an individual who has itch or typical lesions in a typical distribution

*Notes*:

1. *These criteria should be used in conjunction with the full explanatory notes and definitions (in preparation)*.
2. *Diagnosis can be made at one of the three levels (A, B or C)*.
3. *A diagnosis of Clinical and Suspected scabies should only be made if other differential diagnoses are considered less likely than scabies*.

The advantage of the levels and categories is that the criteria may be applied in a range of situations and settings. For example, a clinical trial of new therapies may choose to only enrol participants with Confirmed Scabies (level A) as this would be the most specific and least sensitive level. Alternately, for field mapping, Clinical and Suspected Scabies (levels B and C) may be used, acknowledging these would be less specific but more sensitive. Where examination of genital areas is not feasible or appropriate, due to a lack of visual privacy or other dignity considerations, sub-category B2 could be omitted. Investigators using the criteria are advised to list the distribution of diagnoses by sub-category, which will further allow comparison between survey findings.

Whilst these criteria are not intended to replace the clinical acumen and assessment of clinicians treating individual patients, we anticipate there may be clinical applications, particularly as a reference in settings where non-expert health workers are required to make a diagnosis. Clinical practice requires consideration of other factors outside of these criteria, such as history and progression over time, and the benefits and risks of treating or not treating individuals. Therefore, the threshold for diagnosis and treatment may be higher or lower in some cases.

Although this Delphi process resulted in high agreement for the diagnostic criteria, there are several limitations. In the absence of an appropriate reference standard test, or adequate data on the accuracy of clinical criteria, this process is reliant on expert opinion, which may not correlate with diagnostic accuracy. Therefore, an important next step is the validation of the criteria in a range of settings, including different geographic regions, areas of differing prevalence, and where other important differential diagnoses may be prevalent.

These criteria should be viewed as a starting point, allowing consistency and comparison in collected data. The criteria are likely to require revision, for example, if accurate laboratory or point-of-care tests for scabies infection become available. Although the criteria allow for diagnosis in mapping scenarios using a quick skin examination, the inclusion of history features, particularly relating to contact history, is not ideal for rapid assessment. Therefore, further research into the accuracy and utility of more simplified approaches to assessment are needed. It may be that more simplified assessment (for example, a limited skin examination of the limbs) could be used in field surveys, particularly if results can be correlated back to a comprehensive skin examination including assessment of itch and contact history.

Application of the criteria, summarized in Box 1, requires reference to the evidence-based explanatory notes and definitions that are being prepared in parallel. These notes will clearly define and explain each feature and subcategory of the criteria and will include recommended techniques for examination, microscopy and visualization, as well as details of important differential diagnoses. To enable use of the criteria for epidemiological mapping, a training methodology will be required for non-expert health workers, similar to that developed for trachoma graders [17].

Strong consensus has been established for criteria to diagnose scabies in a variety of settings. These criteria will facilitate diagnosis and comparison of findings across studies. The 2018 criteria require validation in a range of epidemiological settings.

## Supporting information

S1 Data Deidentified dataset of the Round 4 survey (Table 3). (XLSX)
